# Unmasking Bacillus Calmette–Guérin Immune Reconstitution Inflammatory Syndrome in a Perinatal HIV Transmission—A Case Report

**DOI:** 10.3390/tropicalmed10060148

**Published:** 2025-05-23

**Authors:** Daniel Ivanov, Dimitar Strashimirov, Rusina Grozdeva, Evgeni Penchev, Elena Georgieva, Nina Yancheva

**Affiliations:** 1 Department of Infectious Diseases, Parasitology and Tropical Medicine, Medical University-Sofia, 1431 Sofia, Bulgaria; d.strashimirov@medfac.mu-sofia.bg (D.S.); r.grozdeva@medfac.mu-sofia.bg (R.G.); n.yancheva-petrova@medfac.mu-sofia.bg (N.Y.); 2 Department of AIDS, University Hospital for Infectious and Parasitic Diseases “Prof. Ivan Kirov”-Sofia, 1431 Sofia, Bulgaria; evgeny.penchev@gmail.com; 3 Clinic of Pediatric Pneumology and Phthisiology, University Hospital “St. Ivan Rilski”, 1431 Sofia, Bulgaria; bronchi@abv.bg

**Keywords:** BCG vaccination, HIV, IRIS, BCG lymphadenitis

## Abstract

Bacillus Calmette–Guérin (BCG)-related immune reconstitution inflammatory syndrome (IRIS) is a recognised complication following antiretroviral therapy (ART) initiation in HIV-infected infants. We report the case of a 19-month-old child with undiagnosed perinatally acquired HIV due to maternal nondisclosure. The child developed ipsilateral axillary lymphadenitis at the BCG vaccination site shortly after starting ART. The clinical features and temporal association with ART supported a diagnosis of BCG-IRIS. The child was successfully managed with conservative pharmacological treatment alone—rifampicin, isoniazid, and macrolide therapy—without surgical incision or corticosteroids. Progressive improvement of the lesion was observed, and complete clinical resolution occurred over the following months, alongside immune recovery. This case underscores the importance of recognising BCG-IRIS, even in settings where HIV diagnosis may be delayed, and supports the feasibility of conservative management in paediatric patients, potentially avoiding surgical intervention in settings of localised disease.

## 1. Introduction

BCG-related complications in HIV-infected children are unusual in Europe and North America, with reports more commonly coming from tropical and subtropical regions [1,2]. They can, however, arise as immune reconstitution inflammatory syndrome (IRIS) following antiretroviral therapy (ART) initiation. Despite growing awareness of BCG-IRIS in paediatric HIV, there remains no universally endorsed treatment protocol [3,4]. Management strategies vary widely—from observation to antimycobacterial therapy and, in some cases, surgical intervention—and are largely drawn from HIV-uninfected populations [1,5]. Importantly, no randomised controlled trials (RCTs) have evaluated the effectiveness of antibiotic or surgical therapies specifically for BCG-induced disease in HIV-infected infants [5]. This limited and indirect evidence base complicates clinical decision making. Here, we describe a case of BCG-IRIS in an HIV-infected infant successfully managed with a conservative, non-surgical approach.

## 2. Case Report

The patient—a 19-month-old child—was born at term via vaginal delivery following an uneventful second pregnancy. All mandatory neonatal immunisations, including BCG, were administered at birth. Although the mother had reportedly tested HIV-negative during antenatal screening, further investigation revealed a prior diagnosis nearly a decade earlier. She had not engaged in care and treatment, consciously withheld her status during the pregnancy, and circumvented routine HIV prevention protocols. As a result, her status remained undisclosed to the obstetric team. The child was breastfed for two months and developed normally until 12 months of age, when signs of psychomotor regression were noted. Routine bloodwork showed anaemia and thrombocytopenia. A paediatric neurologist confirmed truncal hypotonia and loss of developmental milestones. Elevated transaminases were also documented. The child was hospitalised in a tertiary centre for further evaluation. The workup showed cytopenias, abnormal liver function tests (LFTs), hepatosplenomegaly, and generalised lymphadenopathy upon imaging, raising concern for a lymphoproliferative disorder. Brain MRI (magnetic resonance imaging) revealed bilateral subcortical T2 hyperintensities and nonspecific gliotic changes, interpreted as perinatal in origin. Bone marrow aspiration showed trilineage haematopoiesis, dyserythropoiesis, and histiocytic activation without malignancy. Due to the constellation of progressive neurological symptoms, haematological abnormalities, and hepatosplenomegaly, infectious disease screening was pursued. HIV infection was confirmed at the National Centre of Infectious and Parasitic Diseases through two consecutive fourth-generation ELISA assays, followed by Western blot and, finally, PCR (polymerase chain reaction) testing. Afterward, the child was referred to the Acquired Immunodeficiency Unit in Sofia for ART initiation and ongoing care.

Upon admission to the ward, the child appeared moderately unwell but afebrile. Examination also revealed generalised lymphadenopathy and hepatosplenomegaly. The rest of the general physical examination was unremarkable. Neurological examination revealed increased lower-limb tone, hyperreflexia, and positive Babinski and Rossolimo signs—presentation consistent with HIV encephalopathy in infancy. Labs showed pancytopenia, elevated LFTs, and a normal C-reactive protein (CRP). A chest radiograph showed clear lung fields without focal infiltrates, cavitation, hilar lymphadenopathy, or radiographic evidence of primary pulmonary tuberculosis. Based on the neurological presentation, the child met the WHO (World Health Organization) clinical criteria for stage IV disease, consistent with HIV encephalopathy. This diagnosis was further substantiated immunologically, as the child’s CD4 count was 137 cells/μL (3%), which is well below the < 25% threshold used to define advanced HIV infection in children aged 12–35 months. HIV viral load was 439,732 copies/mL. Given the child’s WHO stage IV classification, ART was initiated promptly with dolutegravir (DTG), zidovudine (AZT), and lamivudine (3TC), alongside trimethoprim/sulfamethoxazole prophylaxis. Although abacavir (ABC) is the European AIDS Clinical Society (EACS)-preferred first-line agent, the unavailability of paediatric ABC and tenofovir alafenamide in Bulgaria necessitated the use of AZT/3TC, as the only NRTI backbone compatible with DTG. On day six post-ART, the child developed a fever of 38.5 °C without any other clinical signs. A comprehensive respiratory PCR panel for twelve common respiratory pathogens yielded a negative result.

**Figure 1:**
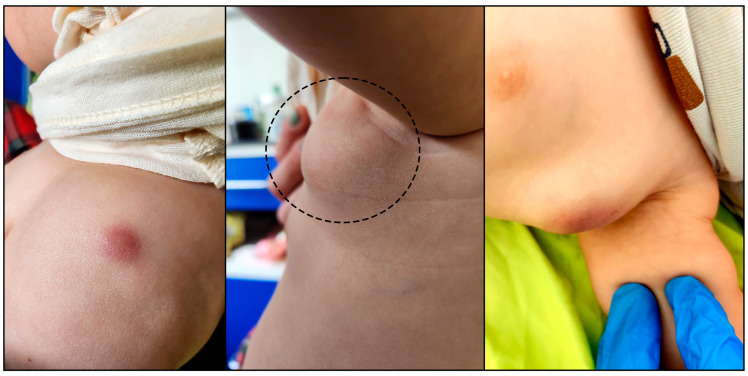
**Left**: Inflamed Bacillus Calmette–Guérin (BCG) vaccination scar in the left deltoid region, presenting as a well-defined erythematous lesion. The inflammation developed shortly after ART initiation and coincided with the clinical onset of axillary lymphadenitis. **Middle**: Initial lesion (outlined with a dashed line) in the left axillary region, photographed two days after presentation, showing a firm, non-fluctuant, mildly erythematous swelling consistent with early BCG lymphadenitis. No signs of suppuration or overlying skin breakdown were observed at this stage. **Right**: Same axillary site a few days later, showing increased swelling, skin discoloration, and early liquefaction suggestive of suppurative lymphadenitis.

After 10 days on ART, left axillary lymphadenitis appeared with a 20 mm × 20 mm tender mass, ipsilateral to the BCG scar, which showed inflammation (Figure 1). CRP had spiked to 141 mg/L. Given the temporal association with ART initiation, BCG-IRIS was suspected. Tuberculosis (TB) was considered less likely due to the absence of signs of systemic involvement, a negative T-SPOT.TB, the localised nature of the lymphadenopathy, and no known TB exposures.

Empirical clarithromycin was started for atypical mycobacterial coverage, and ibuprofen for its anti-inflammatory effect. Following pulmonology consultation on day 20 post-ART, rifampicin and isoniazid were recommended as well. Three serial ultrasound-guided assessments performed by paediatric surgeons confirmed two liquefied axillary lymph nodes (19 × 10 mm and 13 × 10 mm), and a conclusion was reached that there was no immediate need for surgical intervention. The lesion improved progressively under conservative therapy. Clarithromycin was switched to azithromycin to reduce drug–drug interactions with rifampicin (a CYP3A4 inducer). As rifampicin lowers dolutegravir serum concentrations, DTG was adjusted to twice-daily dosing. The child was discharged in good general condition after a month’s stay in the clinic.

**Figure 2:**
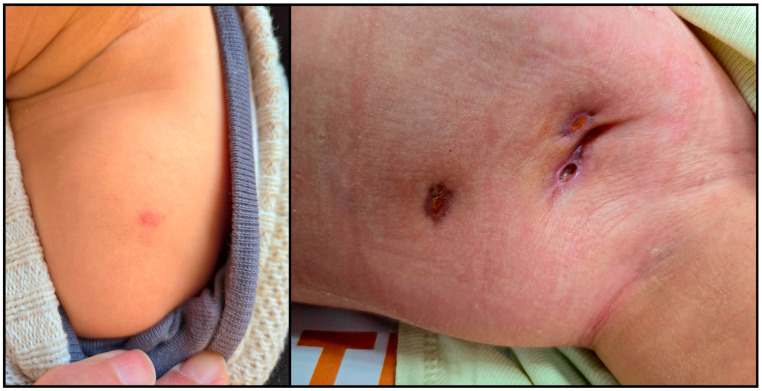
**Left**: Healed BCG vaccination scar in the left deltoid region with residual erythema, following resolution of IRIS. **Right**: Left axillary region showing subsequent healing of the lymphadenitis site, with two well-demarcated epithelializing fistulous tracts.

During a six-month follow-up after discharge, the child underwent multiple evaluations at a paediatric pulmonology clinic. Fistulae developed at the site of the lymph node conglomerate. PCR MTBDR (GenoType Mycobacterium tuberculosis drug resistance assay)—a molecular test used to detect Mycobacterium tuberculosis complex and resistance to rifampicin and isoniazid—was performed on wound secretions and returned a negative result. To further support the diagnosis and rule out active tuberculosis, five serial gastric lavage specimens and two additional wound secretion cultures were obtained for mycobacterial culture and Ziehl–Neelsen staining; none yielded Mycobacterium tuberculosis complex. By the end of the six-month follow-up, the lesion had healed significantly (Figure 2), there was resolution of pancytopenia, and notable neurodevelopmental progress—the child was now able to sit, crawl, and stand with support, alongside improvements in emotional and behavioural functioning. The CD4 count had increased to 574 cells/μL (18%), with a 2.4-log drop in viral load (1771 copies/mL).

## 3. Discussion

The WHO recommends BCG vaccination, which is a part of the majority of national immunisation programmes across the world [6]. Earlier WHO guidelines advised against BCG vaccination in HIV-infected adults and children due to the potential of disseminated BCG disease [7]. More recent updates have re-emphasised the importance of BCG vaccination, despite variations in reported efficacy and effectiveness across different studies and populations. Meta-analyses of RCTs and cohort studies indicate that neonatal BCG vaccination confers 59–82% protection against pulmonary tuberculosis and up to 90% efficacy against severe forms in neonates [8].

In the present case, BCG was administered at birth as per national policy, as the child was not known to be HIV-exposed at the time. Anticipated and internalised stigma likely led the mother to withhold her HIV status and bypass standard antenatal protocols. This resulted in a missed opportunity for prevention and undetected vertical transmission. Such cases are a rare occurrence in Bulgaria, where routine HIV screening in pregnancy is mandatory, and the provision of ART to pregnant women living with HIV has largely eliminated mother-to-child transmission.

Early HIV diagnosis and early antiretroviral therapy reduced infant mortality by 76% and HIV progression by 75% [9]. Rabie et al. evaluated the temporal relationship between ART initiation at different ages and CD4 levels and BCG-IRIS incidence in HIV-infected infants from the CHER (Children with HIV Early Antiretroviral Therapy) trial, and found that initiating ART early—before CD4 decline or clinical progression—reduced the risk of BCG-IRIS by fourfold when compared to deferred ART [10]. Early childhood HIV is marked by an immature and tolerogenic immune system, which hampers effective viral control and drives rapid disease progression; when combined with high post-seroconversion viral setpoints (> 100,000 copies/mL), this greatly increases the risk of AIDS and fatal outcomes [11,12]. In this case, ART was initiated upon diagnosis due to the presence of advanced HIV disease. Perinatally HIV-infected children, especially those with AIDS-defining illnesses, show poorer neurodevelopmental outcomes [13]. Given the documented neurodevelopmental regression in the preceding months, delaying ART posed a risk of further deterioration. The decision to initiate ART promptly in this case is further supported by findings from a cohort study of 74 HIV-infected children (median age of 1.7 years), which demonstrated significant neurodevelopmental gains following ART initiation. After just 6 months of treatment, children exhibited measurable improvements in gross and fine motor function [14]. This parallels the neurodevelopmental progress observed in our patient six months after initiating ART. WHO, EACS, and DHHS (U.S. Department of Health and Human Services on Antiretroviral Therapy and Medical Management of Children Living with HIV) guidelines recommend rapid ART initiation in all HIV-infected children, especially those under five, regardless of CD4 count or clinical stage. While EACS guidelines generally advise delaying ART initiation by up to two weeks in settings of active opportunistic infections to reduce the risk of IRIS, this applies primarily when a treatable infection is identified. Our initial baseline investigations failed to reveal a particular opportunistic pathogen. The child’s deteriorating neurological and immunological status necessitated treatment to preclude further progression and mortality risk. However, with advanced HIV infection and implementation of ART, IRIS may ensue. Besides long-standing or—in the case of children—rapidly progressing infection, low CD4 counts and high HIV-1 RNA at initiation were the strongest independent risk factors for BCG-IRIS [1,10].

In a prospective observational study from sub-Saharan Africa and India, BCG was the leading cause of IRIS, followed by tuberculosis and dermatologic manifestations [1]. BCG-IRIS could be defined as ipsilateral axillary lymphadenopathy (≥10 × 10 mm) occurring within 6 months of ART initiation [10]. Therefore, the HIV-positive infants presenting with ipsilateral axillary adenitis at the vaccination site should raise suspicion for BCG-IRIS [15]. BCG-related complications are typically classified as either regional—near the vaccination site and involving regional lymph nodes—or disseminated, affecting distant sites via haematogenous spread [16]. While disseminated BCG disease remains rare among neonatally HIV-exposed children, regional complications persist, occasionally presenting with marked clinical severity [2,17]. Although the diagnosis is primarily made on clinical grounds [18], mycobacterial infection should still be excluded. According to WHO guidance, TB screening in HIV-positive children under 10 years old involves symptom assessment for cough, fever, poor weight gain, or identifying TB contact. A chest X-ray is strongly recommended to enhance diagnostic sensitivity. CRP may offer added value, especially in ART-naive children, due to its higher sensitivity and specificity than symptoms alone [19,20]. In our case, however, none of these criteria were met. Nonetheless, efforts to rule out mycobacterial disease continued. While gastric lavage cultures can assist in the diagnosis of patients unable to expectorate [21], the diagnostic yield of microbiological cultures is often low with children [1], as evidenced by the repeatedly negative gastric lavage and wound cultures in our patient.

Whereas some BCG-vaccine-induced infections may resolve without therapeutic intervention, BCG lymphadenitis lacks standardised treatment guidelines, particularly in its suppurative forms [3,4,10].

An analysis of five RCTs assessing various interventions (oral, locally instilled antibiotics, and surgical approaches) for BCG-induced disease in children produced inconclusive results. Neither isoniazid, macrolides, nor their combinations (e.g., isoniazid plus rifampicin) demonstrated definitive efficacy [5]. The management of BCG-IRIS with antimycobacterial therapy remains controversial, with prospective and retrospective analyses reporting both conservative approaches and no treatment in many cases [1,10]. Needle aspiration may shorten recovery, although relevant evidence remains limited. While no RCTs have assessed treatment strategies specifically in HIV-infected infants, intervention may be warranted given the potential risks for negative repercussions [5].

Isoniazid monotherapy for 6 months is the standard approach to latent mycobacterial infection or TB prevention in patients on ART [22,23], although rifapentine-based combinations are now recommended where available [19,20]. Alternatively, complete antituberculosis therapy may be initiated under clinical suspicion or suggestive findings of active disease, pending the exclusion of TB [2,20]. In our case, guided by pulmonology consultation, rifampicin and isoniazid were introduced to provide targeted antimycobacterial coverage. Given the high likelihood that Mycobacterium bovis was the causative organism, pyrazinamide was excluded from the outset due to intrinsic resistance. The patient demonstrated an early clinical response to non-steroidal anti-inflammatory drugs (NSAIDs) and clarithromycin, reflected in a fivefold decrease in CRP levels and the resolution of fever, obviating the need for corticosteroids at this stage. This conservative approach aligns with current expert opinion, which supports NSAIDs as effective for managing moderate IRIS symptoms [15]. Notably, corticosteroids such as dexamethasone and prednisolone have been used in severe cases [15]. Although fistulae developed after discharge, this occurred in the context of continued overall clinical improvement and lesion resolution, and it remains uncertain whether corticosteroids would have prevented this outcome [17].

In conclusion, the incidence of serious BCG-IRIS or related disseminated disease is low in high-resource settings with established prevention protocols. However, this case illustrates the rare occurrence of BCG-IRIS linked to delayed HIV diagnosis due to maternal nondisclosure. It also asserts the feasibility of a conservative treatment approach with ART initiation and antimycobacterial therapy without surgery and corticosteroids. Finally, it emphasises the need for further data to better understand and guide the management of BCG-IRIS in perinatally acquired HIV infection.

## Data Availability

The original data presented in this case study are available on reasonable request from the corresponding author. The data are not publicly available due to privacy concerns.
